# Lupus of the Larynx

**Published:** 1886-01

**Authors:** Frank O. Stockton

**Affiliations:** Professor of Laryngology, College of Physicians and Surgeons, Chicago


LUPUS OF THE LARYNX. *By* FRANK O. STOCKTON, M. D. *Professor of Laryngology, College of Physicians and Surgeons, Chicago.*

This disease may well be called one of the curiosities of medicine, both on account of the limited number of cases reported and the scarcity of literature on the subject.

Of the thirteen cases thus far reported, five were contributed by Türck,[1] two each by Tobold[2] and Mackenzie,[3] one each by Ziemssen,[4] Grossman,[5] Lifferts[6] and Beningier.[7]

If the larynx of every subject suffering from cutaneous lupus were examined, Lefferts thinks it would be found more frequently, arguing no doubt from the fact that in most of the cases reported the laryngeal and cutaneous affections were associated.

---

1. Zeitschrift d. Gesellschaft d. Aerzte Zu Wien, 1859. No. 11.
2. Keklkoffkrankhiten, page 307.
3. Diseases of the Throat and Nose. Eng. Ed., page 396.
4. Cyclopædia of Med., N. Y. Ed., VII., page 854.
5. Aug. Wien Med. Ztg. 1877, XX., page 182.
6. Am. Jour. Med. Sci., April, 1878, page 370.
7. Annal. d. Maladies d. l'oreille et du larynx, July, 1878.

The etiology ot lupus has never been clearly settled. Undoubtedly some constitutional defect is the cause. Just what this defect is, is very difficult to say. By some writers it is said to be scrofula. This I am unwilling to admit, as the scrofulous ulcerations that I have met with were of a very different character. I am inclined to think that lupus and tuberculosis are allied very closely.

The symptoms of this disease are in no way diagnostic. In the beginning we have possibly some sore-throat, slight difficulty in swallowing and dyspnœa, there is also hoarseness frequently amounting to complete aphonia. These symptoms are progressive in character as the disease advances.

Examination of the larynx reveals marked changes, but nothing peculiar to the disease; in fact, the many points of resemblance to syphilis, cancer and phthisis, are so marked that a diagnosis can only be made in most cases by exclusion, provided of course there is no cutaneous lesion.

Virchow's[8] description of a case of lupus seen by him will serve as a general description.

"There is an indurated cicatrix studded with thick knobs the size of a pea, extending from the dorsum of the tongue down to its base. The epiglottis was very hard, and bordered with hard warts. From this part the tissues were hardened and warty as far down as the trachea. The arytenoids were deeply ulcerated and surrounded with hard papillary growths."

He further states that the lupus nodules are composed of young and soft granulative tissue, which is usually very vascular. It contains small round cells and originates in the proliferation of the connective tissue and not of the epithelium.

The tendency of the disease is toward destructive ulceration, and in apparant healing instead of a healthy cicatrix, a tissue

8. Thie Krankhaften Geschewülste, II., page 490.

of low vitality is found, which soon becomes the seat of a fresh outbreak.

From what has been previously stated it will be seen that the diagnosis of this disease is by no means easy except it be associated with cutaneous lupus.

In fact, a diagnosis can rarely be made at sight, but only through careful study and treatment.

Cutaneous lupus is considered an intractable disease, so that it would naturally follow that in the laryngeal cavity would be the same.

While not a dangerous disease in itself, the new mouths or warts may be so plentiful that they obstruct the glottis and interfere with respiration.

The action of this trouble is, as a rule, very slow and is occasionally arrested. Experience has shown that surgical and therapeutical means directed against the disease itself have very little or no effect, and that we must treat it through the general system.

The treatment is that usually followed in wasting diseases, viz.: cod-liver oil, and the various tonics. Occasionally caustic applications may be of use, and nitrate of silver appears to give the best results owing to its double action, caustic and stimulant.

To the thirteen cases recorded I wish to add another, making fourteen.

During December, 1884, George L., aged thirty-nine, a shoemaker, came under my care for a slight sore throat and a bad cough.

The patient was very much emaciated and quite pale, and spoke with a peculiar hoarseness. He said he had been troubled with what he thought was catarrh for the past ten or twelve years, but which of late had been getting worse.

On examining the throat it was found that the uvula was very much congested and enlarged, being about one inch long and three-quarter inch wide, and the exterior surface covered with warty growths.

The left palatine arch was very badly eroded, as well as the whole posterior surface of the velum, which was also dotted here and there with the warty growths. The posterior wall of pharnyx, from the base of the nares down to the border of the thyroid cartilage, was ulcerated, with scattered groups of the warty prominences, and coated with a thick yellow tenacious mucus.

The larynx on being examined showed the vocal cords very much congested, but no ulcerations. The arytenoids were deeply ulcerated and surrounded with the warts.

